# PML Surfs into HIPPO Tumor Suppressor Pathway

**DOI:** 10.3389/fonc.2013.00036

**Published:** 2013-03-01

**Authors:** Sabrina Strano, Francesca Fausti, Silvia Di Agostino, Marius Sudol, Giovanni Blandino

**Affiliations:** ^1^Molecular Chemoprevention Group, Molecular Medicine Area, Regina Elena National Cancer InstituteRome, Italy; ^2^Translational Oncogenomic Unit, Molecular Medicine Area, Regina Elena National Cancer InstituteRome, Italy; ^3^Weis Center for Research, Geisinger ClinicDanville, PA, USA; ^4^Mount Sinai School of MedicineNew York, NY, USA

**Keywords:** tumor suppression, pathway, interaction, signaling, transcription

## Abstract

Growth arrest, inhibition of cell proliferation, apoptosis, senescence, and differentiation are the most characterized effects of a given tumor suppressor response. It is becoming increasingly clear that tumor suppression results from the integrated and synergistic activities of different pathways. This implies that tumor suppression includes linear, as well as lateral, crosstalk signaling. The latter may happen through the concomitant involvement of common nodal proteins. Here, we discuss the role of Promyelocytic leukemia protein (PML) in functional cross-talks with the HIPPO and the p53 family tumor suppressor pathways. PML, in addition to its own anti-tumor activity, contributes to the assembly of an integrated and superior network that may be necessary for the maximization of the tumor suppressor response to diverse oncogenic insults.

## The HIPPO Pathway

**Figure 1:**
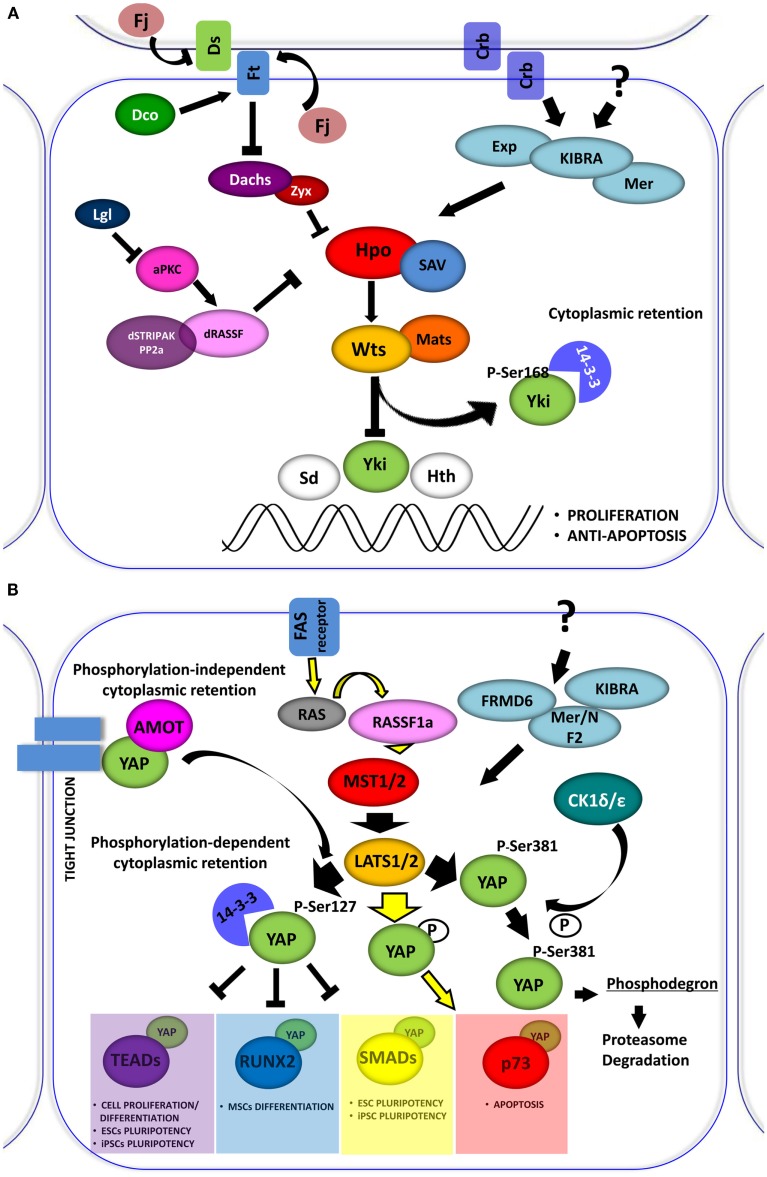
**(A)** The *Drosophila* Hippo pathway. Signaling diagram of Hippo (Hpo) kinases cascade and of its modulation by apical transmembrane protein complexes and proteins involved in cell polarity control (Arrowed or blunted ends indicate activation or inhibition, respectively). **(B)** Role of YAP in the Hpo-like pathway in human. In mammals the relationships between *Drosophila* Hpo and Wts, are conserved in Mst1/2 (Hpo homologs) and Lats1/2 (Wts homolog). Lats1/2 phosphorylates YAP on different conserved motifs. Phosphorylation-dependent 14-3-3 binding and cytoplasmic retention are conserved in YAP, which inhibits it to enhance the transcriptional activation of pro-proliferation genes. Depending on the cellular context Lats1/2 phosphorylates YAP, increasing its transcriptional support to p73 to induce apoptosis. YAP can also be phosphorylated by other kinase, such as CDK1δ/ε promoting its subsequent protein proteasome-degradation. The retention of YAP at cytoplasmic level can also be exerted by sequestering the protein at the junctional level through the interaction with the AMOT protein.

First identified in *Drosophila*, the Hippo (Hpo) signaling pathway is a conserved string of molecular events, which regulates the proper size of organs through the balance of cell growth and cell death. Cell density and contact inhibition information are conveyed by membrane complexes through the Hpo pathway to specific transcriptional programs in the nucleus. In contact-inhibited cells, the Hpo pathway is activated, whereas in sparsely populated cells, it is inhibited. The pathway is characterized by a core kinase, Hpo (Mst1 and Mst2 in mammals), and its downstream effector, Warts (Wts) kinase (Lats1 and Lats2 in mammals), which regulate Yorkie (Yki), protein (YAP and TAZ in mammals), a transcriptional co-activator for transcription factors involved in the induction of cell proliferation, survival, and apoptosis (Figures 1A,B).

As the Hpo pathway plays an important role in organ size control. Loss of function of many individual members of the pathway leads to tumorigenesis. Accordingly, down-regulation of this pathway is frequently associated with human cancers. In addition, the Hpo pathway has been implicated in tissue homeostasis through the regulation of stem cells, cell differentiation, and tissue regeneration.

### The *Drosophila* Hippo pathway

The “blueprint” for the Hpo pathway emerged from genetic screens of *Drosophila*, Wts was the first identified Hpo pathway member (Justice et al., 1995), encoding a kinase of the Nuclear Dbf-2-related (NDR) family. Several years later, four tumor suppressors, named Hpo, Wts, Salvador (Sav), and Mats were implicated in the regulation of organ size in flies (Figure 1A). These proteins constitute the core kinase cassette of the pathway, whose products can affect proliferation without increasing susceptibility to apoptosis (Justice et al., 1995; Tapon et al., 2002; Wu et al., 2003; Lai et al., 2005). Wts activity, which is fundamental for the phosphorylation-dependent regulation of Yki (Huang et al., 2005; Oh and Irvine, 2008), is regulated through a series of phosphorylation events. Wts is directly phosphorylated by Hpo and this phosphorylation is facilitated by Sav protein (Tapon et al., 2002; Wu et al., 2003). Hpo is a member of the Sterile-20 family of Ser/Thr kinases. It interacts with and phosphorylates Sav, which in turn activates Hpo kinase function. Mob as tumor suppressor (MATS) is similar to Sav and acts as an adaptor protein. Mats also belongs to the NDR family and binds Wts, potentiating its intrinsic activity (Lai et al., 2005). Hpo phosphorylates Mats, increasing its binding affinity to Wts and potentiating Wts kinase activity (Praskova et al., 2008).

Located upstream of the Hpo pathway are Merlin (Mer) and Expanded (Ex; Boedigheimer et al., 1993; LaJeunesse et al., 1998; Hamaratoglu et al., 2006). These are considered tumor suppressors that cooperate with each other to control organ growth. Kibra, a newly discovered member of the Hpo pathway, is involved in formation of a Kibra-Mer-Ex, which constitutes an apical protein complex required for the association of the Hpo pathway with cellular membranes (Genevet et al., 2010; Yu et al., 2010). Crumbs (Crb) is a transmembrane protein involved in determining cell polarity. The loss of Crb expression was shown to further determine a phenotype characterized by overgrowth (Chen et al., 2010; Ling et al., 2010; Robinson et al., 2010). It is likely that Crb is one of the membrane receptors that regulate the Hpo pathway. Other proteins involved in cell polarity control have been linked to the Hpo pathway as well, including: Scribble (Scrib), Disk large (Dlg), Lethal giant larvae (Lgl), and a typical protein C (aPKC; Chen et al., 2010; Grzeschik et al., 2010). The atypical cadherin FAT (Ft) was the first transmembrane protein shown to affect Hpo signaling and was the first tumor suppressor gene isolated in *Drosophila*. Complete knock-out of the Ft protein induced cell death in *Drosophila* larvae with overgrown imaginal disks (Mahoney et al., 1991). Many lines of evidence suggest that the principal mechanism exerted by Ft is on the Wts function (Feng and Irvine, 2007, 2009). Many Ft partners were identified as potential modulators of the Hpo Pathway: (i) Dachsous (Ds), an atypical cadherin which binds to Ft (Matakatsu and Blair, 2004, 2006); (ii) Four-jointed (Fj), a kinase that typically localizes to the Golgi subcellular compartment (Ishikawa et al., 2008); (iii) a Casein I kinase, termed Dco (Disks overgrown), responsible for Ft phosphorylation in its cytoplasmatic segment (Cho et al., 2006; Feng and Irvine, 2009); and (iv) Dachs, which is an unconventional myosin that antagonizes Ft (Cho et al., 2006). All of these components are believed to be responsible for linking Hpo to extracellular stimuli (Harvey and Tapon, 2007).

Yorkie, a critical effector of the Hpo pathway, is not a direct transcriptional factor but is a potent transcriptional co-activator cooperating with different DNA binding proteins. Wts phosphorylates Yki at Ser 168, thus creating a binding site for 14-3-3 proteins, which in turn sequester Yki in the cytoplasm and prevent its nuclear import (Dong et al., 2007b; Oh and Irvine, 2008). Loss of Hpo signaling, as well as mutations in the 14-3-3 binding site for Yki, were shown to produce strong nuclear accumulation coupled with enhanced activity of Yki (Zhao et al., 2007). Interestingly, some binding partners of Yki are the same kinases that function upstream to it in the Hpo pathway (e.g., Ex, Wts, and Hpo; Badouel et al., 2009; Oh et al., 2009). Other partners are represented by transcription factors that govern different classes of genes. One class includes genes involved in cell survival and proliferation: scalloped (Sd), a member of TEAD/TEFs family (Wu et al., 2008; Zhang et al., 2008), and Homothorax (Hth), both of which promote cell proliferation (Peng et al., 2009). Sd, together with Yki, up-regulate transcription of *Diap1* gene, an apoptosis inhibitor (Wu et al., 2008). Hth is involved in regulating the transcription of another Yki target, the growth promoting microRNA gene, *bantam*. Other Yki targets in this class are the cell-cycle regulators *Cyc E*, *E2F1*, and *dMyc* (Drosophila Myc; Goulev et al., 2008; Peng et al., 2009; Neto-Silva et al., 2010). The second class of genes are components of other signaling pathways including Notch, Wnt, EGFR, and Jak-Stat pathways. For example, Smad proteins are functional partners of Yki, potentiating the transcriptional response to BMP/TGF-β signaling (Alarcon et al., 2009). Finally, a third class of Yki targets consists of several proteins from its own Hpo cascade, including Ex, Mer, Kibra, Crb, and Fj (Cho et al., 2006; Hamaratoglu et al., 2006; Genevet et al., 2009, 2010).

### The mammalian Hippo pathway

The Hpo pathway is evolutionarily conserved in mammals (Figure 1B). It was reported that loss of function in mutant flies can be rescued by expressing their respective human counterparts (Wu et al., 2003; Lai et al., 2005). One ortholog for the adaptor protein Sav, termed WW45 or SAV1, and two orthologs for Mats, termed MOBKL1A and MOBKL1B have been identified. These proteins form a conserved kinase cassette that phosphorylates and inactivates the mammalian Yki homologs, YAP and TAZ, preventing their nuclear translocation and consequently attenuating the transcription of genes involved in proliferation and differentiation (Huang et al., 2005; Zhao et al., 2007; Lei et al., 2008).

MST1/2 are serine-threonine kinases, which are able to initiate apoptosis when overexpressed (Lin et al., 2002; Ura et al., 2007). MSTs become activated by auto-phosphorylation on threonine residues within their activation loop domain. The inhibition of dimerization and auto-phosphorylation of MST2 exerted by the proto-oncogene serine/threonine protein kinase RAF1 was reported (O’Neill et al., 2004). In this latter context, expression of the scaffold protein RASSF1A is able to release MST2 from this inhibition, thus inducing apoptosis (Matallanas et al., 2007). Moreover, it was shown that PP2A phosphatase dephosphorylates MST1/2 kinases, thus inhibiting their function (Ribeiro et al., 2010). MST substrates include LATS and MOB1. LATS1/2 kinases control cellular homeostasis, negatively regulating cell division cycle 2 (CDC2) and favoring G2/M arrest (Yang et al., 2001; Xia et al., 2002; Yabuta et al., 2007). LATS2 was also reported to induce G1/S arrest (Li et al., 2003). The loss of LATS1/2 was shown to lead to a broad variety of tumors, such as soft tissue sarcoma and leukemia (St John et al., 1999). These proteins are strong tumor suppressors. LATS activity is supported by MOB1, which binds to and activates LATS kinases, favoring YAP and TAZ proto-oncogene phosphorylation and inhibiting their nuclear activity.

The mammalian genome contains orthologs for most of the reported upstream regulators of the Hpo pathway in flies. Notably, it encodes more than one paralog for most of the *Drosophila* Hpo components. Two homologs were identified for Kibra: KIBRA/WWC1 and WWC2, as well as for Ex: FRMD6 and FRMD1, while only one homologous was identified for Mer: NF2. Paralogs for MST and LATS kinases are well characterized and Yki orthologs, YAP and TAZ exhibit similar function in regulating signals related to cell growth or apotosis. The presence of conserved *Drosophila* orthologs in the mammalian Hpo pathway may very well serve as a mechanism of redundancy that protects the organism against cancer-causing mutations.

## YAP: A Critical Component of the Hippo Pathway

Yes-associated protein (YAP) was identified as Yes (and Src) kinase-interacting protein (Sudol, 1994; Sudol et al., 1995). The complex between YAP and Yes was recently validated functionally in colon cancer cells by the Bill Hahn laboratory (Rosenbluh et al., 2012). Originally, YAP was shown as a transcriptional co-activator that binds the PPxY motif in Runx1 (or AML1 for acute myeloid leukemia 1; Chen and Sudol, 1995; Yagi et al., 1999). Soon after, it was reported that YAP, like its paralog TAZ, binds 14-3-3 in a phospho-dependent manner (Kanai et al., 2000) and YAP plays a role as a co-activator for the TEAD/TEF family of transcription factors (Vassilev et al., 2001; Basu et al., 2003). The conserved Hpo pathway kinase cassette triggers the phosphorylation and inactivation of YAP, preventing its nuclear import and consequently attenuating the transcription of genes regulating proliferation (Huang et al., 2005; Zhao et al., 2007; Lei et al., 2008).

At least eight different splicing isoforms of YAP exist (Komuro et al., 2003; Gaffney et al., 2012). YAP orthologs in lower organisms, such as *Drosophila*, have tandem WW domains, indicating that the double WW domain organization is conserved from the fly to humans. The WW is a small modular protein domain that has two conserved Tryptophan (W) residues (Bork and Sudol, 1994; Sudol et al., 1995). The WW domain shares a similar function with the SH3 domain, able to bind short peptides that are Proline-rich. Initially, the WW domain was shown to bind to Proline-rich peptides terminated with Tyrosine (Y), so called PY motifs (Chen and Sudol, 1995; Einbond and Sudol, 1996; Sudol and Hunter, 2000). YAP has been found to interact with various proteins of diverse functions and the majority of these interactions occur through the YAPs WW domains (reviewed in Bertini et al., 2009; Sudol, 2010, 2012; Sudol and Harvey, 2010). Apart from WW domains, the modular structure of YAP contains a TEAD factor-binding domain (TB), which is located at the amino-terminal portion of the protein (Vassilev et al., 2001), a carboxy-terminal SH3 binding region, transcriptional activation domain and PDZ-binding motif, which is critical for nuclear translocation and indispensable for binding the PDZ motif of ZO-1 and ZO-2 (Oka and Sudol, 2009; Oka et al., 2010). None of the signaling pathways known is as rich in WW domain-containing proteins as the Hpo pathway (Sudol and Harvey, 2010). The intact WW domains of YAP are required for their co-transcriptional activator function and promoting cell proliferation (Zhao et al., 2009).

The activation of the Hpo pathway triggers the inhibition of YAP activity (Figure 1B). YAP is phosphorylated by LATS on different serine residues, among them, serine 127 appears to be the most critical for its function (Basu et al., 2003). This phosphorylation generates a 14-3-3 binding site on YAP, which induces its cytoplasmic retention, thus inhibiting its co-transcriptional activity (Basu et al., 2003; Zhao et al., 2007; Hao et al., 2008). YAP is also phosphorylated by LATS on Ser 381 (Zhao et al., 2010). The Ser 381 modification primes YAP to subsequent CK1δ/ε phosphorylation and degradation via the SCF (beta-TRCP) E3 ubiquitin ligase complex.

Hippo pathway activation is also triggered by cell–cell contacts. High-cell density induces YAP phosphorylation and cytoplasmic retention (Zhao et al., 2007) and the disruption of cell–cell junctions in epithelial tissue, induces nuclear translocation of YAP and TAZ (Varelas et al., 2010).

There are phosphorylation-independent mechanisms through which YAP could be sequestered in the cytoplasm. It was observed that YAP was able to interact with the angiomotin (AMOT) family of proteins. This interaction occurs via the WW domains of YAP and sequesters the protein in the cytoplasm and junctional complexes (Chan et al., 2011; Wang et al., 2011; Zhao et al., 2011). The existence of a tripartite complex between AmotL1, YAP, and ZO-2, which regulates YAP function, apparently in opposite directions, was highlighted recently (Oka et al., 2012). The cytoplasmic retention of YAP, exerted by AmotL1, disabled YAP in its nuclear function, whereas ZO-2 enhanced the nuclear translocation of YAP (Oka et al., 2012).

Yes-associated protein serves as a co-regulator for various transcriptional factors, including members of the TEAD family factors, Smad proteins, RUNX, Erb4, and p73 (reviewed in (Sudol, 2010; Mauviel et al., 2012). Among these targets, only the interaction with the TEFs/TAEDs appears to be conserved from flies to humans (Vassilev et al., 2001; Wu et al., 2001; Mahoney et al., 2005; Sawada et al., 2008). Two major target genes for YAP-TEADs complexes were described in mammals: *CTGF* and *Cyr61* (Lai et al., 2011). *CTFG* is a direct target of YAP-TEADs complex and was shown to be critical for YAP-induced proliferation and anchorage-independent growth. Another YAP target is *Myc*. Similar to the *Dropsophila* Yki-Sd effect on *dMyc* expression (Neto-Silva et al., 2010), YAP was shown to up-regulate the expression of *Myc* gene in transgenic mouse liver (Dong et al., 2007a). Therefore, it is not surprising that the targeted disruption of the transcriptional complex of YAP-TEADs by genetic approaches or pharmacological compounds is the main focus of drug discovery efforts aimed at cancers with amplified YAP oncogene (Liu-Chittenden et al., 2012; Sudol et al., 2012).

As the transcriptional co-activator of SMAD proteins, YAP seems to also be involved in embryonic stem cell (ESC) pluripotency. The maintenance of this ability by ESC is governed by coordinating multiple pathways, including TGFβ and BMP pathways. Both YAP and TAZ were shown to be critical mediators of both these pathways. YAP interacts physically with SMADs, driven by TGFβ and BMP toward their transcriptional function (Varelas et al., 2008, 2010; Alarcon et al., 2009; Sudol, 2012). The ESC pluripotency is described as the ability to give rise to all types of cells and tissues maintained in adults. Thus, differentiated cells can also be reprogrammed in ESC-like cells, termed induced pluripotent stem cells (iPSC), by activating a given set of transcription factors. iPSC cells can then self-renew and differentiate again in every type of cell and tissue. This ability is due to the activity of distinct transcription factors such as KLF4, cMYC, OCT4, and SOX2 (Takahashi and Yamanaka, 2006). SMADs proteins were shown to co-occupy the same genomic site of OCT4 and SOX2 in human ESC (Mullen et al., 2011). Moreover, YAP was shown to favor, possibly on a transcriptional level, reprogramming mouse embryonic fibroblasts in an iPSC-like state (Lian et al., 2010). The majority of genomic sites for ESCs transcription factors also contain a consensus for TEF/TEADs transcriptional binding factors, suggesting that YAP could be implicated in this mechanism also through its interaction with TEADs. Interestingly, it was observed that simultaneous knocking down of TEAD1, TEAD3 and TEAD4 inhibits the ESC pluripotency in mice (Lian et al., 2010).

Many lines of evidence indicate that the Hpo pathway may play an important role also in mesenchymal stem cells (MSC), which are able to differentiate into osteoblasts, adipocytes, chondrocytes, and myoblasts. Consistent with this observation is the discovery of another important YAP transcriptional partner, RUNX2, which is essential for skeletal mineralization because it stimulates osteoblast differentiation of MSC, promotes chondrocyte hypertrophy, and contributes to endothelial cell migration and vascular invasion in bone development. Like other RUNT-domain proteins, RUNX2 is a context-dependent transcriptional activator and repressor of genes that regulate cellular proliferation and differentiation. YAP was shown to interact with full-length RUNX2 in osseous cells via co-immunoprecipitation of endogenous proteins and co-immunofluorescence (Zaidi et al., 2004). RUNX2 recruits YAP to subnuclear foci and to the osteocalcin gene promoter, but does not affect its nucleo-cytoplasmic shuffling. The signature Y residue in the PPPYP motif of RUNX2 is essential for interacting with YAP (Chen and Sudol, 1995; Yagi et al., 1999). YAP-mediated repression of RUNX2 activity on the osteocalcin promoter is cell-type independent. YAP-mediated repression of RUNX2 instead seems to be dependent on the promoter context: YAP blocked RUNX2-dependent activation of *TGF*β*R1* promoter and enhanced RUNX2-dependent repression of its own promoter; however it did not affect RUNX2 transcriptional effects on the *p6OSE2* or *p21* promoters. These data indicate that RUNX2 can recruit YAP to promoter regions, but the effects of YAP on the expression of RUNX2 target genes is dependent on the cohort of other DNA binding proteins and co-factors brought to the gene by specific DNA sequences and protein–protein interactions. Transcriptional repression of RUNX2 by YAP depends on Src-induced activation and tyrosine phosphorylation of YAP (Zaidi et al., 2004). Dominant-negative Src and YAP proteins, as well as Src kinase inhibitors, increased RUNX2 transcriptional activation of the osteocalcin promoter in ROS 17/2.8 cells (Zaidi et al., 2004). The idea that YAP could support RUNX2 function as co-activator has been confirmed by a recent study in which authors observed that a nuclear localized mutant form of YAP can induce osteogenesis. It was observed that YAP is part of a signaling complexes that forms its response to mechanical signals exerted by extracellular matrix (ECM) rigidity and cell shape. This regulation requires Rho GTPase activity and tension of the actomyosin cytoskeleton, but is independent of the canonical Hpo pathway. YAP is required for differentiation of MSC induced by ECM stiffness and for survival of endothelial cells regulated by cell geometry (Dupont et al., 2011).

## PML and p53: A Close Link in Tumor Suppression

In humans, a complete or partial loss of PML expression has been observed in multiple types of cancers, including breast, colon, and prostate (Gurrieri et al., 2004a,b). PML KO mice develop normally, but are resistant to lethal doses of γ-IR. In addition, they are prone to tumorigenesis in response to carcinogens, or an additional oncogenic event, such as the loss of PTEN (Wang et al., 1998; Trotman et al., 2006). PML is a key factor in the formation of PML nuclear bodies (PML-NBs), which are distinct nuclear multi-protein complexes that have been associated with critical cellular processes, including tumor suppression, gene regulation, post-translational modifications, and protein catabolism. The stabilization of PML protein levels leads to recruitment of p53 to the PML-NBs, an event that facilitates p53 acetylation and transcriptional activation (Pearson et al., 2000; Bernardi and Pandolfi, 2007). It was demonstrated that in conditions of oncogenic stress, PML is a p53 transcriptional target gene (de Stanchina et al., 2004). PML contains three putative p53 responsive elements (REs) in its promoter region (de Stanchina et al., 2004). p53 is able to induce PML protein as well as PML mRNA, and to increase the number and size of PML-NBs. PML also contributes to cellular p53-dependent processes like senescence, cell-cycle arrest, and p53-mediated apoptosis, and additionally PML arranges p53 tumor suppressor functions (de Stanchina et al., 2004). Recently, it has been reported that PML expression is also regulated at the translational level during oncogenic K-RAS-induced OIS in a p53-independent manner (Scaglioni et al., 2012). The authors show that mTOR-dependent translational control mechanisms are important in modulating PML protein levels during oncogenic K-RAS-induced OIS and that the PML 5′ untranslated mRNA region plays a key role in mediating PML protein upregulation and OIS induction (Scaglioni et al., 2012). Haupt and colleagues found that PML interacts and co-localizes with mutant p53. PML activates mutant p53 transcriptional activity and is important for its gain of function in cultured human cancer cells. These findings support the notion that as is the case for wtp53, PML is a key regulator of mutant p53 (Haupt et al., 2009).

## p73/YAP and PML/YAP: Two Cooperative Pro-Apoptotic Protein Complexes

**Figure 2:**
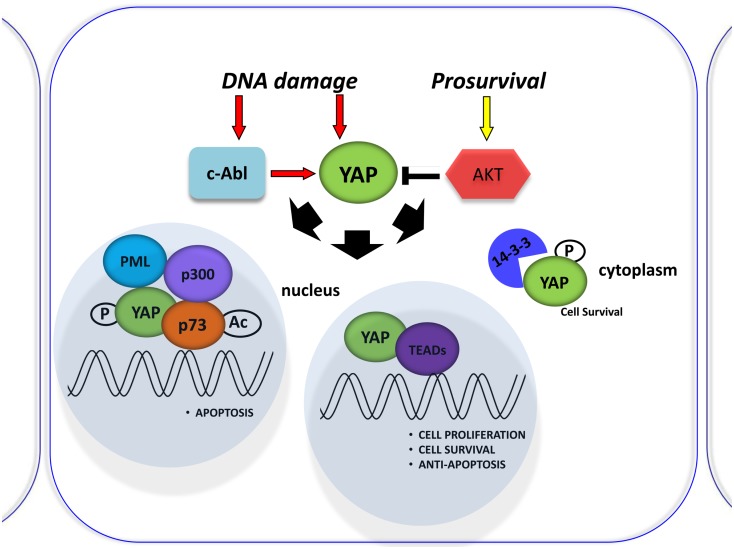
**Involvement of YAP in the DNA damage response**. Schematic representation of the DNA damage response induced by Cisplatin (CDDP) treatment. It was shown to signal c-Abl-mediated YAP phosphorylation, resulting in YAP nuclear localization and increased p73 binding and activation of pro-apoptotic genes. By contrast, active Akt counters this effect by phosphorylating YAP and sequestering it at the cytoplasmic level.

p53 protein is known as the “guardian of the genome” and is the focus of studies in understanding tumorigenesis. Another member of the p53 family, p73, was recently discovered for its pivotal role in DNA damage signaling. The main activities of the p53 family occur through the transcriptional activation or repression of target genes that encode key proteins involved in cell growth inhibition, apoptosis, senescence, and differentiation (Vousden and Lu, 2002). However, the members of the family differ in their upstream regulation by different kinases. The YAP is a critical mediator of p73 function. It binds p73 to regulate its transcriptional activity (Strano et al., 2001) and subsequent cell death induction (Basu et al., 2003). This binding is negatively regulated by Akt-mediated YAP phosphorylation (Basu et al., 2003; Figure 2) and enhanced by DNA damage (Strano et al., 2005). Furthermore, YAP stabilizes p73 protein in a post-translational way by competing with the ITCH E3-ligase for binding to p73 (Levy et al., 2007) and inducing its transcriptional activity via the p300 acteyltansferase (Strano et al., 2005). Rossi et al. (2005) have shown that Itch, a human ubiquitin protein ligase that belongs to the Nedd4-like E3 family containing a WW domain, binds, and ubiquitinates p73 and determines its rapid proteosome-dependent degradation.

The promyelocytic leukemia protein (PML) has been shown to bind p73 in the nuclear bodies (NBs) to promote p300-mediated acetylation of p73 and to regulate its transcriptional activity (Bernassola et al., 2004). PML is the key component of PML-NBs that regulate a large number of cellular processes by facilitating post-translational modification of target proteins, promoting protein–protein contacts, or by sequestering proteins. It functions as a tumor suppressor, required for normal, caspase-dependent apoptosis in response to DNA damage, FAS, TNF, or interferons. It plays a role in transcription regulation, DNA damage response, DNA repair, and chromatin organization (Salomoni and Pandolfi, 2002; Bernardi and Pandolfi, 2007; Alsheich-Bartok et al., 2008). Therefore, Blandino’s group described the PML tumor suppressor protein as a mediator of the YAP-p73 complex (Strano et al., 2005). Furthermore, they demonstrated that PML was a direct transcriptional target of p73/YAP, showing that PML transcriptional activation by p73/YAP is under the negative control of the proto-oncogenic Akt/PKB kinase (Figure 2). Importantly, PML and YAP physically interact through their PVPVY and WW domains. This direct interaction provides part of the mechanism by which PML can enhance the activity of the YAP-p73 complex (Lapi et al., 2008). Binding assays and site-specific mutagenesis have shown that the PY “XPPXY” motif consensus binds with relatively high affinity to the WW domain of YAP (Chen and Sudol, 1995; Sudol et al., 1995). The PVPVY motif of PML is not a canonical consensus sequence for YAP WW domain binding but the VPxY peptide, along with LPxY, SPxYs peptides were shown to be exceptions that were able to bind to YAP WW domain (Chen and Sudol, 1995; Chen et al., 1997). This evidence may be important in suggesting the possibility of interactions of YAP with a plethora of proteins, which contain non-canonical WW domain binding sequences and participating in various cellular and metabolic pathways. Together with the specificity of the cellular contexts in which YAP binds to its canonical or non-canonical partners, these observations may also explain the dual role of YAP as both an oncogene and tumor suppressor.

It has also been demonstrated that PML contains a SUMO binding motif that is independent of its sumoylation sites necessary for PML-NBs formation (Shen et al., 2006). According to a large number of proteins associated with the PML, NBs are sumoylated, and/or contain SUMO binding motifs. This suggests the possibility that these proteins are recruited to the PML networks through non-covalent interactions mediated by covalently bound SUMO and SUMO binding motifs present in PML. Furthermore, YAP was identified as a target for PML-mediated sumoylation that blocks YAP polyubiquitylation and its subsequent degradation. This could also be important in mediating the recruitment of YAP/p73 complex to the PML-NBs (Lapi et al., 2008). In fact, the stabilized YAP in the protein complex with p73, is then recruited to PML-NBs to achieve full transcriptional activation of p73. The spatial and temporal kinetics of the recruitment and competition for binding must be elucidated to have a more complete picture of this dynamic complex. All these views contribute to the idea that YAP/PML complex could act as a bridge at the crossroads of many signaling pathways, where the actual biological read-out might lay in the cellular context of binding partners, including cell-specific transcription factors or effectors (apoptosis, DNA damage response, senescence, cancerogenesis). In any case, it is important to stress the importance of post-translational modifications in the regulation of YAP activity. Phosphorylation is the most common described modification of YAP, but as demonstrated by Lapi and colleagues, different afferent signals may modulate the choice of the partners during physiological cell life and the onset of cancer.

## The Transcriptional Axis YAP/PML/p73 in DNA Damage-Induced Apoptosis

The regulation of p73 activity by YAP has mainly been investigated in the field of DNA damage signaling. As an activating cofactor for a pro-apoptotic transcription factor, one line of thinking has assumed that YAP plays a tumor suppressor role in cancer and cell culture models (Oka et al., 2008; Yuan et al., 2008). However, YAP has also been clearly identified as an oncogene and cell size regulator in both *Drosophila m*. and mammalian cells (Dong et al., 2007b; Zhao et al., 2007; Cordenonsi et al., 2011; Halder et al., 2012).

Lapi et al. (2008) demonstrated that active Akt counters cisplatin-induced increases in PML transcription via the YAP-p73 complex. Cisplatin (CDDP) was shown to signal c-Abl-mediated YAP phosphorylation, resulting in YAP nuclear localization and increased p73 binding and activation of pro-apoptotic genes (Levy et al., 2008; Figure 2). Those investigating the role of YAP phosphorylation events downstream of cisplatin have so far focused on its subcellular localization for its effect on p73 binding, but perhaps different phosphorylations can affect YAP stability (Levy et al., 2007). However, the molecular mechanism was not described. The upregulation of YAP protein, upon CDDP treatment, is not due to transcriptional regulation, but is a post-translational event that correlates with an increase in YAP sumoylation mediated by PML, since it is completely abrogated in PML/MEFs. Elevation of YAP levels in response to DNA damage demonstrates another level of regulation of this pathway, suggesting that p73 activation must be tightly controlled to ensure quick and efficient activation of p73 target genes in response to stress conditions (Lapi et al., 2008).

The last findings demonstrate the existence of a positive regulatory loop between the p73/YAP protein complex and PML during apoptosis triggered by CDDP in HCT116 cells (Lapi et al., 2008).

**Figure 3:**
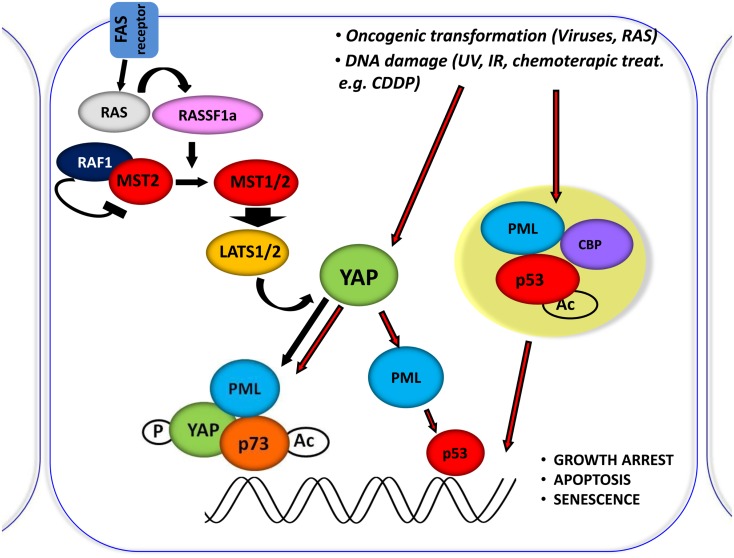
**Central role of YAP in proliferation inhibiting pathways**. Schematic representation of the central role of YAP in inducing the inhibition of proliferation, by integrating and mediating different upstream stimuli. It was shown that RASSF1A disrupts the inhibitory complex between RAF1 and MST2 and favors the physical association between MST2 and LATS1 concomitantly; therefore, leading to YAP1 phosphorylation and nuclear re-localization where it binds to p73 and potentiates its apoptotic activity. Oncogenic transformation as well as DNA damage (achieved by CDDP treatment) leads to YAP accumulation and to its functional activation.

In fact PML, YAP, and p73 can be recruited on Bax and p53AIP1 apoptotic gene promoters, which contain p73 binding sites within their promoter regions, in response to CDDP and PML binds to its own promoter and first intron where YAP and p73 were also recruited, and promotes its own transcriptional activation (Figure 3).

It has been previously demonstrated that YAP requires PML and NBs localization to coactivate p73 (Strano et al., 2005). We describe that p73 and YAP are required not only for the transcriptional activation of PML during the apoptotic response but also for the subsequent accumulation of PML protein and formation of NBs. As a result, PML can contribute to the p73-dependent apoptotic response by promoting both p300-mediated acetylation of p73 (Bernassola et al., 2004) and stabilizing YAP by inhibiting its ubiquitin-mediated degradation.

Whether YAP-p73 regulation of PML gene transcription or PML regulation of the YAP-p73 complex is signaled by other stresses besides cisplatin, needs to be addressed in the future.

Based on what we have previously described, YAP is not a bona-fide tumor suppressor, but a transcriptional co-activator that impinges directly or indirectly on different tumor suppressor pathways, here represented by p53 family, PML, and HIPPO (Figure 3). As for transcription factors with tumor suppressor function, YAP could also reinforce either by binding directly or indirectly the activity of transcription factors whose gene targets encode for oncogenic proteins. Further studies of when and how YAP is stabilized and to which transcription factor complexes it is recruited will help us understand the paradoxical activities of YAP in DNA damage and tissue growth, as well as its contradictory roles in tumorigenesis in different tissues.

## Concluding Remarks

Our work has revealed a direct crosstalk between PML and YAP, one of the two main effectors of the Hpo tumor suppressor pathway. This finding has several important ramifications for our understanding of the molecular mechanism of cancer. A good example comes from a recent study of glioblastoma multiforme (GBM), one of the most aggressive human tumors (Okazaki et al., 2012). The authors showed that the level of PML protein in human glioma tissue decreased as the degree of malignancy increased. A complete loss of PML was observed in 11.1% of GBM biopsies. The report described solid evidence that after the treatment of TMZ (temozolomide)-resistant glioma cells with both interferon-β (IFN-β) and TMZ, the expression of the endogenous PML gene and its associated protein increased. This effect was primarily attributable to IFN-β, which activates and translocates p73/YAP complex into the nucleus for interaction with PML to induce glioma cell apoptosis, explaining in part the observed *in vivo* anti-tumor effects. The knowledge of precise mechanisms underlying the activation of the endogenous PML/p73/YAP axis is essential to induce the apoptosis of specific cancer cells, in addition to the DNA damage inflicted by the combination therapy.

We hope that the unexpected finding of “PML surfing into the YAP-Hpo tumor suppressor pathway” will allow us to better understand the entire Hpo signaling *network*, and to design systems biology approaches to fight cancers caused by the genetic lesions that map within, and affect the network.

## Conflict of Interest Statement

The authors declare that the research was conducted in the absence of any commercial or financial relationships that could be construed as a potential conflict of interest.
